# Vegetable-Oil-Loaded Microcapsules for Self-Healing Polyurethane Coatings

**DOI:** 10.3390/polym17233184

**Published:** 2025-11-29

**Authors:** Efterpi Avdeliodi, Sofia Derizioti, Ioanna Papadopoulou, Aikaterini Arvaniti, Kalliopi Krassa, Eleni P. Kalogianni, Joannis K. Kallitsis, Georgios Bokias

**Affiliations:** 1 Department of Chemistry, University of Patras, 26504 Patras, Greece; chem3577@ac.upatras.gr (E.A.); up1066326@ac.upatras.gr (S.D.); up1073739@ac.upatras.gr (I.P.); up1087065@ac.upatras.gr (A.A.); kallitsi@upatras.gr (J.K.K.); 2 Megara Resins Fanis S.A., 38th KM New National Rd. Athens-Corinth, 19100 Megara Attikis, Greece; p.krassa@megararesins.com; 3 Department of Food Science and Technology, International Hellenic University, 57400 Thessaloniki, Greece; elekalo@ihu.gr

**Keywords:** microcapsules, vegetable oils, olive oil, soybean oil, castor oil, polyurethane coating, self-healing, anti-corrosion

## Abstract

Smart self-healing polymer materials are breaking open new pathways in industry, minimizing waste, and enhancing the long-term reliability of applications. Moreover, when they possess anti-corrosive properties, they effectively protect surfaces from wear and corrosion, leading to improved and more robust products. In the present work, we develop a series of new self-healing polyurethane coatings activated by temperature, through the encapsulation of vegetable oils (VO), namely olive, soybean, and castor oil, in the core of polyurea microcapsules (VO-MCs). Using a green method, water-dispersible microcapsules were embedded in water-based polyurethane matrices. Both the self-healing ability and the anti-corrosive properties of the respective films were evaluated after mechanical damage. Encapsulation allowed for the direct release of VOs into the damaged area; subsequently, the temperature increase reduced the viscosity of the oils, facilitating their flow and diffusion into the damaged area and accelerating the healing process. Soybean oil and olive oil showed remarkable performance in terms of self-healing and high anti-corrosion ability for the polyurethane coatings, while castor oil showed a limited anti-corrosion effect but quite satisfactory effectiveness in terms of self-healing. Overall, the study highlights the potential of using encapsulated oils in environmentally friendly, active coatings with dual action: corrosion protection and self-repair of damage.

## 1. Introduction

Microencapsulation plays a key role in the design of new materials, which is useful for a wide range of applications. Microcapsules are micron-sized objects, composed of a shell (usually polymeric) and a (liquid) core. The active compounds are encapsulated in the core, while the shell protects the compounds from environmental factors and, eventually, allows for a stable and controlled release from the capsule core [1]. Microencapsulation has, thus, been widely investigated in areas such as food [2], cosmetics [3], personal care, textiles [4], agriculture [5], material development [6], etc. The food and cosmetics or personal care industries have put particular emphasis on the encapsulation of different types of oils in polymer microcapsules, with a focus on essential oils. Compounds, which release fragrance into the environment, are encapsulated or embedded in the polymeric microcapsules, giving the product a pleasant aroma, flavor, and/or a fatty consistency. Examples of the most used natural oils are citrus oils (e.g., orange, mandarin, or lemon), seed oils (e.g., anise, cumin, or cardamom), fruit peel oil (e.g., cinnamon) or root oils (e.g., ginger or turmeric) [7]. Encapsulation provides chemical stability to these materials, since such compounds are sensitive to light, heat, or oxygen [8] and may deteriorate during the synthesis and storage until the final application by the end-user [9].

Natural oils as a general category, including essential oils, have a lot of other uses, such as antimicrobial [10], anticorrosive [11,12], or self-healing [13] materials. Metal corrosion has long been a significant challenge in everyday life. A commonly adopted method to overcome the effects of corrosion is to use polymeric coatings, which delay the corrosion procedure on the metallic surface [14,15]. However, polymeric coatings are systems that are susceptible to damage under mechanical or environmental stress. As a result, they could show superficial or deep cracks and damages, resulting in their malfunction and a reduction in their lifetime [16]. To face this problem, the class of self-healing coatings has evolved as a promising solution in recent years. Self-healing coatings can be self-repaired without any human intervention [17]. An extrinsic self-healing mechanism relies on external healing agents, like microcapsules or vascular networks, that release a restorative substance upon the damage. In contrast, intrinsic self-healing uses a material’s own reversible chemical bonds and physical interactions to repair itself without external additives [18]. The most common examples of self-healing systems are coatings containing nano/microcapsules, loaded with healing agents. Oil-in-water emulsion polymerization is applied as a facile and effective technique for the development of capsules with encapsulated compounds [19]. Design development is crucial, as recent studies show that preparation technology directly influences both their structure and functionality. Parameters such as agitation rate, viscosity, and monomer concentration quantitatively determine size, shell thickness, and encapsulation efficiency. It has also been established that minor differences in synthesis conditions can significantly modulate the mechanical properties and release rate of encapsulated composites, highlighting the direct relationship between preparation methods and the design of high-performance materials. Similar correlations have been reported in recent studies of polymer coatings, which systematically examine the impact of synthesis conditions on anti-corrosion and self-healing behavior [20,21,22].

Upon cracking, the healing agents are released from the capsules and form a new coating in the damaged area [23]. Among others, drying oils like linseed oil [24] or tung oil [25] are proposed as effective, sustainable healing agents. Drying oils are vegetable oils characterized by a large proportion of carbon–carbon double bonds—these are able to polymerize and harden when exposed to air, forming a solid film. This process occurs through oxidative polymerization of the unsaturated fatty acids [26,27], making VOs ideal core materials for self-drying under atmospheric conditions, with no need for a catalyst [24].

The technology of encapsulated drying oils has found extensive application in epoxy coatings [27,28,29], exploring a broad range of polymeric materials for shell formation, such as urea-formaldehyde [30,31,32], polyurethane [33,34], and polyurea [35]. On the other hand, the studies concerning the incorporation of such microcapsules into polyurethane coatings are comparatively less common [36,37] and focus mainly on highly reactive drying oils, like linseed and tung oil. Therefore, the encapsulation of alternative oils, such as olive oil, soybean oil, or castor oil in polyurea shells and their subsequent dispersion in waterborne polyurethane coatings, is an under-explored approach, offering an opportunity to significantly expand the range of functional MCs-PU systems [38,39,40]. The most commonly studied types of microcapsules in this case feature polyurethane or polyurea shells and encapsulate within their core active agents, such as low molecular weight diisocyanates (e.g., isophorone diisocyanate, IPDI, or hexamethylene diisocyanate, HDI) [41,42,43], catalysts [44], or other functional compounds that are capable of repairing mechanical damage in the polymer matrix.

The formation of the polyurea shell has been elaborated in our previous works [45,46], where the monomer methylene diphenyl diisocyanate (MDI) reacts easily and rapidly with diethylenetriamine (DETA), forming a 3D polyurea network under mild reaction conditions. This allowed for the effective encapsulation of IPDI, acting as the releasable self-healing agent of waterborne polyurethane coatings (WPUs) [47]. WPUs have become particularly important in self-healing coating applications, as they combine environmental friendliness, high flexibility, and the ability to include “smart” healing mechanisms. The use of water instead of organic solvents reduces volatile organic compound (VOC) emissions [48], while their structure (with soft and hard segments [49]) offers mechanical strength and elasticity, facilitating bond reconnection after damage. In addition, the polar character of WPUs enables functional modification and makes it possible to develop sustainable “smart” coatings with self-healing properties [50,51,52].

Based on this experience, the preparation and characterization of vegetable-oil-loaded microcapsules (VO-MCs) for similar applications are described in the present work. The encapsulation of three natural products, such as olive oil, castor oil, and soybean oil, has been attempted, applying oil-in-water emulsion polymerization as a common and effective technique for the development of the major goals, which are the design of the synthesis and characterization protocols for effective encapsulation, as well as the elucidation of anticorrosion and self-healing abilities of the synthesized VO-MCs, when embedded in WPUs coatings.

## 2. Materials and Methods

### 2.1. Materials

Poly(vinyl alcohol) (PVOH, Mw = 130,000 with 88% hydrolysis degree), methylene diphenyl diisocyanate (MDI), diethylenetriamine (DETA), cyclohexanone, acetone, and sodium chloride were obtained from Merck (Darmstadt, Germany). Olive oil (extra virgin) and soybean oil (refined) were obtained from a local store, while castor oil (refined) was obtained from a local pharmacy. Hexane and chloroform were obtained from Alfa Aesar (Ward Hill, MA, USA). Ultrapure water was prepared using an Arium mini water purification system (Sartorius, Göttingen, Germany). All chemicals in this study were used as received, without additional purification. WPUs (waterborne polyurethane dispersions) have the following characteristics: (i) NCO/OH ratio of 1.4, (ii) non-volatile content (NVC) of 36 wt.%, (iii) pH of 7.1, (iv) acid value of 27.3 mg KOH/g and solvent content of ~8 wt.%. They are based on commercial polycarbonate polyol (MW 1000 g/mol, OH value ~110 mg KOH/g, f = 2).

### 2.2. Synthesis of VO-MCs

The organic phase was formed by dissolving 1 g MDI into 4 mL cyclohexanone under magnetic stirring at room temperature. After dissolution, the desired amount of vegetable oil (olive oil, castor oil, or soybean oil) was added to the isocyanate solution and left under magnetic stirring until a macroscopically homogeneous solution was obtained. The organic phase was then mixed with the emulsifier phase (40 mL of a 2.5 wt.% aqueous PVA solution) and homogenized for 2–3 min at 4000 rpm under room temperature conditions (RT). Finally, 7.5 mL of an aqueous 0.5 M DETA solution was added to obtain the microcapsule shell. Emulsion polymerization was allowed to proceed for 3 h at 4000 rpm at RT. The microcapsules were collected after filtration using filter paper, washed twice with ultrapure water, and left to dry at RT for two days. The characterization of the capsules was performed by attenuated total reflectance—Fourier transform infrared spectroscopy (ATR-FTIR), optical microscopy (OM), and scanning electron microscopy (SEM). Table 1 summarizes the synthesis of O-MCs, C-MCs, and S-MCs, corresponding to microcapsules loaded with olive, castor, and soybean oil, respectively.

The amount of oil in the organic phase ($P_{Feed}$ wt.%) was defined by Equation (1).

(1) $$P_{Feed}=\left(\frac{{Moil}_{Feed}}{{\Sigma Morganic\;phase}_{Feed}}\right)\times 100,$$

where ${\Sigma Morganic\;phase}_{Feed}$ is the sum of the masses of MDI, cyclohexanone, and vegetable oil in the feed and ${Moil}_{Feed}$ is the mass of added oil.

### 2.3. Extraction of Encapsulated Oil (wt.%) from VO-MCs

For O-MCs and S-MCs: Microcapsules were crushed in an agate mortar and subjected to ultrasonication (Bransonic 1510E-MT, Branson/Gemini BV, Apeldoorn, The Netherlands) in hexane for 60 min. The supernatant was centrifuged (CAPP CR-656, TechnoLab, Athens, Greece) at 6500 rpm for 10 min. This step was repeated three times. The resulting suspension was filtered, and the precipitate (MC’s shell) was collected and dried under a vacuum at 50 °C. The UV-Vis spectra of the supernatants (hexane solution with released oil) were recorded in the wavelength range of 200 nm–500 nm.

For C-MCs: A similar procedure was followed, using chloroform as the organic solvent. The UV-Vis spectra of the supernatants (chloroform solution with released oil) were recorded in the wavelength range of 200 nm–500 nm, and the ATR–FTIR spectrum of the precipitate (MC’s shell) and supernatant (chloroform solution with released oil) were recorded in the wavelength range of 400–4000 cm^−1^. Measurements on the ATR-FTIR instrument were performed using evaporation of the volatile solvent (in this case, chloroform). A drop of the supernatant solution was placed on the instrument’s diamond and left for sufficient time to allow for solvent evaporation, before recording the ATR-FTIR spectrum.

### 2.4. Calibration Curves and Quantification of Encapsulated Oil (wt.%) in VO-MCs

The calibration curve for olive oil in hexane was established by plotting the absorption (A) at 270 nm [53] versus olive oil concentrations (wt.%), Figure S1. Similarly, for the construction of the calibration curve of soybean oil in hexane, the absorption (A) at 268 nm [54] was plotted versus soybean oil concentrations (wt.%), Figure S2. Linearity ranges of 0.1–1.6 wt.% for olive oil and 0.1–1.0 wt.% for soybean oil were obtained using the proposed approach.

The UV-Vis spectra of the extracted VO–hexane mixtures were initially recorded in the 200 nm–400 nm wavelength range. In most cases, the oil content of the supernatant was high, and the UV-Vis spectra were saturated in the 200 nm–290 nm range. Therefore, to apply the calibration curves and to determine the concentration, Cd, of the diluted sample, the dilution of the mixtures by the desired dilution factor, W, was necessary, permitting the determination of the VO concentration of the original mixtures, Cst, using Equation (2):

(2) $$C_{st}=W\;C_{d},$$

The concentration of the stock solution (Cst) is the oil content (wt.%), which indicated the amount of olive oil (${Moil}_{ext}$) in the mass of the organic solvent (Msolvent). The mass of the solvent was equal to the product of the volume of the solvent and its density.

(3) $${Moil}_{ext}=\frac{C_{st}}{Msolvent},$$

The mass of olive oil (${Moil}_{tot}$) in the total mass of capsules ($m_{MCs\;tot}$) was given by the following relation:

(4) $${Moil}_{tot}=\frac{m_{MCs\;tot}}{m_{MCs\;ext}}{Moil}_{ext},$$

where $m_{MCs\;ext}$ is the MCs extracted mass.

The actual entrapment (P) of the oil in the microcapsules was calculated using Equation (5):

(5) $$P=\left(\frac{{Moil}_{tot}}{{\Sigma Morganic\;phase}_{Feed}}\right)\times 100,$$

### 2.5. Preparation of Coatings

Polyurethane films were prepared via film casting of VO-MCs/WPUs mixtures. A specific amount of 10 wt.% aqueous dispersion of microcapsules (VO-MCs) was added to the aqueous dispersion of the selected WPU. The final VO-MCs content of films MC_solid_/PU_solid_ was 3 wt.%, where MCs_solid_ and PU_solid_ were the values of the solid amount of microcapsules and polyurethane, respectively. The final mixtures were transferred on a Teflon sheet or on a steel surface and left to dry at room temperature until the full evaporation of the solvent and the formation of the film or coating. The thickness of the coatings was approximately 100–150 μm.

On the occasion of metallic coating developments, the metal surface used was low-carbon steel. These substrates were rectangular plates (with a length of 15 cm, a width of 7 cm, and a thickness of 1 mm) and were mechanically polished with SiC sandpaper to achieve a smooth surface. The metals were then cleaned with ethanol and deionized water and dried before the anti-corrosion coating/test was applied.

### 2.6. Self-Healing Tests

The self-healing WPU coatings containing 5 wt.% VO-MCs (based on the polyurethane mass) were investigated. The VO-MCs mother solution (a 10 wt.% aqueous dispersion) was prepared by dispersing the required amount of microcapsules in water to achieve a final concentration of 5 wt.%. The films were left at room temperature (RT) until the solvent had completely evaporated over a period of five days. Scratches were made on the surface of the neat polyurethane coating and the self-healing film. The scratched surfaces were observed immediately after scratching, as well as after 48 h, using a Nikon Eclipse L150 optical microscope (and Nikon’s NIS-Elements DS-U3 software) (Nikon metrology, Paris, France). The healing process was thermally activated by placing the scratched films at 60 °C overnight or by adding a few drops of water to the film’s surfaces. The next day, the scratched films stayed at room temperature.

### 2.7. Anticorrosion Protection Tests

Anticorrosion protection tests were carried out on the self-healing coating and neat polyurethane coating to assess the difference in corrosion resistance. A scratch is applied to a metal surface coated with the self-healing coating material or the pure coating material. A scalpel was used to make scratches on the coated metals (15 cm × 7 cm). The width of the scratch is about 380 μm, and the length of each scratch is 10 cm. Two scratches draw the letter X on each metal. The metal surfaces are left for 48 h at 60 °C. A few drops of the aqueous 5 wt.% NaCl solution (a methodology often described as the “neutral salt drop test”) was placed on the scratch at a temperature of 25 ± 2 °C to investigate the anticorrosion property of the VO-MCs-loaded coatings. Changes in the metal surface were visually observed under the neutral salt environment (NSE). On each metal, coated or not, a neutral salt drop was placed to the lower right corner of the metal, serving as a blank anticorrosion test (red circle).

### 2.8. Characterization

Attenuated total reflection—Fourier transform infrared spectroscopy (ATR-FTIR) spectra were collected using an Alpha II spectrometer (Bruker) (Billerica, MA, USA), with a diamond ATR crystal. All spectra were recorded in the range of 4000–400 cm^−1^, with an average of 34 scans and a spectral resolution of 4 cm^−1^. The morphology and size distribution of the obtained MCs were determined through scanning electron microscopy (SEM), using a Zeiss ZUPRA 35 VP-FEG instrument (Jena, Germany), operating at 5–20 keV. An aqueous dispersion of MCs was spread on a Si wafer, allowed to dry, and coated with a conductive Au film through sputtering. The ultraviolet–visible spectroscopy (UV-Vis) spectra at (200–500) nm were recorded using a Hitachi U-1800 UV−Vis spectrophotometer (Dallas, TX, USA) equipped with a circulating water bath, set at 25 °C. A Nikon Eclipse L150 optical microscope (and Nikon’s NIS-Elements DS-U3 software) was used to evaluate the self-healing properties of the VO-MCs-loaded polyurethane films.

### 2.9. Determination of Isocyanate Content

According to the ASTM D2572 [55,56] standard, isocyanate functional groups—NCO—of the samples were transformed into the urea using dibutylamine solution in toluene, followed by the titration of the excess amine with standardized hydrochloric acid solution [57]. All organic solvents were dry to avoid the reaction between NCO groups and residual water. The relative percentage of the NCO compound can be calculated from Equation (6). Titrations were performed in triplicate, providing a mean error of ±2%.

(6) $$NCO,\%=\frac{\left(V_{blank}-V\right)\times {Ν}_{HCl}\times 0.042}{W}\times 100,$$

where NCO, % is the NCO content in organic solvent and $V_{blank}$ (mL) and V (mL) are the volumes of the standard HCl aqueous solution consumed during the blank experiment and sample titration, respectively. ${Ν}_{HCl}$ is the normality of standard HCl aqueous solution and 0.042 is the milliequivalent weight of the NCO group.

## 3. Results

### 3.1. Synthesis and Characterization of VO-MCs

**Figure 1:**
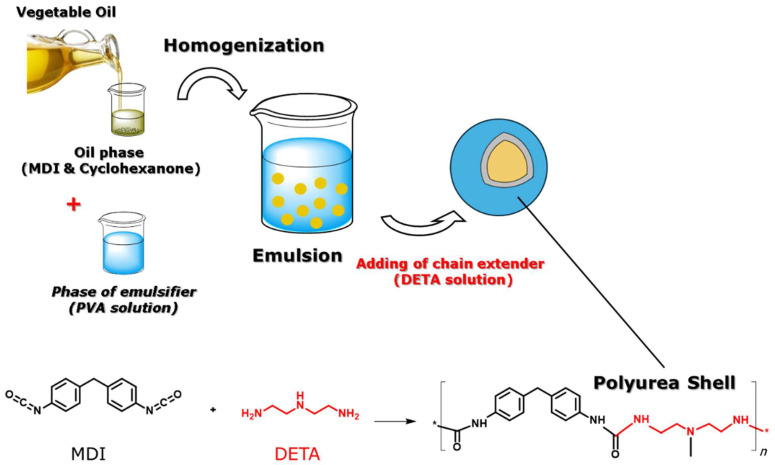
Schematic illustration of the preparation of VO-MCs. The symbol (*) schematically indicates continuation of the polymeric chain.

The preparation of the VO-MCs is achieved by interfacial oil-in-water emulsion polymerization, as shown in Figure 1. The organic phase consists of the organic solvent (cyclohexanone), the monomer (MDI), and the desired vegetable oil. The emulsion is formed through mixing the organic phase with the aqueous phase, containing PVA as an emulsifier, at 4000 rpm. During emulsification, PVA forms a cross-linked network at the interface between the emulsifier phase and the organic phase [58,59]. The polyurea shell of MCs is formed through the interfacial reaction of MDI and DETA (added dropwise in the emulsion), encapsulating the vegetable oil in the core.

**Figure 2:**
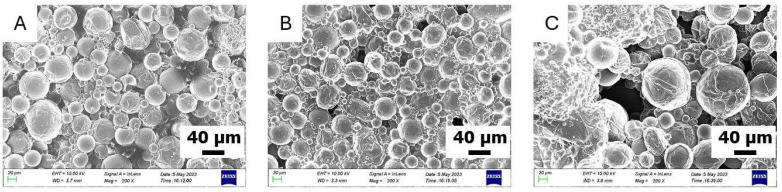
SEM images of microcapsules of (**A**) O-MCs(50), (**B**) O-MCs(60), and (**C**) O-MCs(70).

The oils used in the present work are olive oil, castor oil, and soybean oil (Figure 2). All three oils are of a vegetable source, as they are obtained—after processing—from the fruits or seeds of the respective plant. Olive oil is obtained from the olives of the olive tree (Olea europea) and consists of a mixture of triacylglycerols. The fatty acid composition of olive oil is oleic acid (53–83%), linoleic acid (3.5–21%), palmitic acid (7.5–20%), stearic acid (0.5–5%), and α-linolenic acid (0–1.5%). Here, virgin olive oil is used, which has a content of free (non-esterified) fatty acids of up to 2% [60]. The composition varies depending on the variety, region, altitude, harvest period, and extraction process. Soybean oil is extracted from soybean seeds. Its mixture of triacylglycerols consists of linoleic acid (51%), oleic acid (23%), α-linolenic acid (7–10%), palmitic acid (10%), and stearic acid (4%). Finally, castor oil is produced from the beans of castor, namely the tropical plant, Ricinus communis. Regarding the composition of castor oil, it is also a mixture of triacylglycerols and consists of ricinoleic acid (85–95%), oleic acid (2–6%), linoleic acid (1–5%), α-linolenic acid (0.5–1%), stearic acid (0.5–1%), palmitic acid (0.5–1%), and dihydroxystearic acid (0.3–0.5%). This oil has many hydroxyl groups (-OH), which make the oil chemically active and suitable for many applications [61,62,63].

All encapsulation attempts are summarized in Table 1. Aiming at oil-rich MCs, for all oils, we started with high feed compositions, $P_{Feed}$. The microcapsules are denoted as O-MCs(x), S-MCs(x) and C-MCs(x), when the encapsulated oil is olive oil, soybean oil and castor oil, respectively, while x corresponds to the designed $P_{Feed}$ in each case.

Concerning O-MCs, three successful syntheses have been carried out to encapsulate quite high contents of olive oil in the organic phase, namely 50, 60, and 70% wt. This is readily discernible, as the morphology and size distribution of the capsules O-MCs(50), O-MCs(60), and O-MCs(70) can be evaluated by SEM (Figure 2). As seen, the shearing conditions applied for the MCs preparation (4000 rpm) do not significantly affect the integrity of MCs, in line with previous studies [45,46]. Moreover, the characteristics of the synthesized capsules are not considerably influenced by the wt.% of olive oil content in the organic phase. The particles formed in all cases have a relatively spherical shape, with a non-uniform distribution of microcapsule sizes. In many instances, the shells show fragments of additional layers, which are not complete.

The diameter of the microcapsules is in the range of (8–80) μm for O-MCs(50) and O-MCs(60), while O-MCs(70) are somewhat larger, since now the diameter of the microcapsules is in the range of (20–115) μm.

For further characterization of the microcapsules, ATR-FTIR spectra were recorded. Figure S3A shows the spectra of O-MCs(50), O-MCs(60), and O-MCs(70), as well as the spectra of MDI and olive oil, for comparison. The peaks at 2925, 2850, and 1457 cm^–1^ are attributed to the C-H bonds of the hydrocarbons of the oil, while the peak at 1743 cm^–1^ is attributed to the C=O bond of the triglyceride of the oil (light blue area). These peaks are also observed in the spectrum of olive oil. Moreover, the characteristic peaks of polyurea are identified (1639 cm^–1^, attributed to C=O, and 1542 cm^−1^, attributed to NH), confirming the synthesis of the polyurea shell (pink area). Finally, the strong peak at 2287 cm^–1^ is assigned to unreacted NCO groups of MDI (pink arrow).

**Figure 3:**
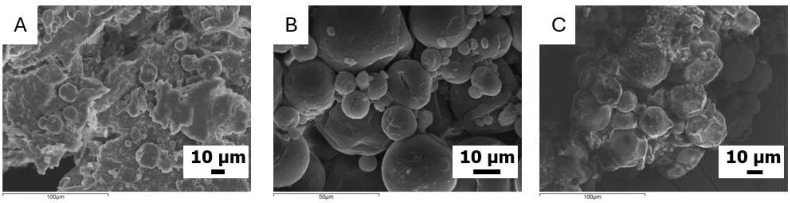
SEM images of microcapsules of (**A**) S-MCs(40), (**B**) S-MCs(50), and (**C**) S-MCs(60).

In the case of the S-MCs containing soybean oil in the organic phase of the emulsion, our attempts were not as successful as previously, as the dimensions of the synthesized capsules were found to be significantly lower (Figure 3). In fact, for the first attempt (S-MCs(40)), where the organic phase contains 40 wt.% soybean oil, a bulk material was mainly obtained. A small number of dispersed spherical particles with a size range of (5–27) μm in diameter is observed. Moreover, for the S-MCs(50) attempt, mostly spherical microcapsules are observed. The diameter of the microcapsules is found within the range of (3–50) μm. Finally, for the S-MCs(60) attempt, with the highest content (60 wt.%) of soybean oil in the organic phase, aggregates of microcapsules with an irregular shape are mostly observed. The size of the few spherical microcapsules observed is found within the (3–37) μm range. Similar bands to those observed in O-MCs are also recorded in the ATF-FTIR spectra of the S-MCs (Figure S3B): namely, the bands attributed to the oil and the polyurea structure, as well as the peak, corresponding to unreacted NCO groups.

**Figure 4:**
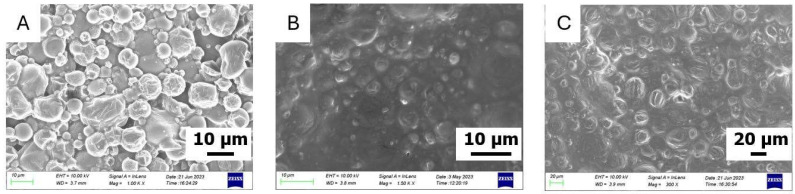
SEM images of microcapsules of (**A**) C-MCs(20), (**B**) C-MCs(30), and (**C**) C-MCs(40).

As this concerns castor oil, five trials have been attempted to prepare the respective microcapsules (C-MCs) with 20, 30, 40, 50, and 60 wt.% content of oil in the organic phase. For the attempts with the higher oil contents, 50 and 60 wt.%, the emulsions were not stable, and precipitation occurred just 5 min after homogenization. For lower oil contents (20, 30, and 40 wt.%), the emulsions were sufficiently stable, allowing for interfacial polymerization to take place. The materials obtained (C-MCs(20), C-MCs(30), and C-MCs(40)) were evaluated by SEM (Figure 4). As seen in the case of C-MCs(20), the microcapsules have a spherical morphology with relatively smooth surfaces. There is some variation in size, but the capsules are quite distinct and clearly observable. The structure shows good homogeneity and cohesion. The size of these microcapsules is within the (3–15) μm diameter range, which is relatively smaller than the S-MCs. The samples C-MCs(30) and C-MCs(40) appear to be more «pressed” and less clearly separated from each other. The surface is not as smooth, but appears more “cloudy” and uneven. It is possible that the increase in the percentage of castor oil affects the stability of the wall and leads to the aggregation or deformation of the surface. Although not examined in detail herein, the different performances of the different oils when forming emulsions could be connected to their composition, which affects the interfacial activity and droplet coalescence. In fact, soybean oil has small amounts of surface-active compounds, while virgin olive oil contains free fatty acids (in the order of 2%). In contrast, castor oil presents increased polarity, as the major component is ricinoleic acid, containing a hydroxyl group.

The characteristic peaks of the oil, as well as the characteristic peaks of the polyurea shell, are also recorded in the ATF-FTIR spectra (Figure S3C) of C-MCs. However, it is worth noting that the characteristic peak of unreacted NCOs at 2287 cm^–1^ (pink arrow) is much smaller in the case of C-MCs compared to that observed for O-MCs and S-MCs. In fact, this peak decreases as the content of castor oil in the organic phase increases. The first explanation is that the OH groups present in the chemical structure of castor oil may react with the excessive NCO groups, in this case.

### 3.2. Determination of Encapsulated Oil in VO-MCs

#### 3.2.1. Olive and Soybean Oil

It is critical to quantify the proportion of the active oil entrapped in the core of the microcapsules: namely, the quantity of oil remaining that is free to migrate to the damage when the shell is disrupted. A key point to note is that the oils are not further oxidized during the production of emulsions or until the absorption measurements are recorded.

**Figure 5:**
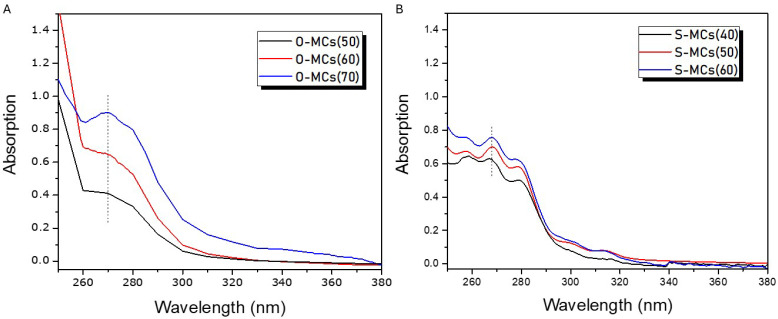
UV-Vis spectra of extracted core for (**A**) O-MCs and (**B**) S-MCs. The vertical dashed line indicates the wavelength selected for each oil, which is used for the corresponding calculations.

To this end, an extraction procedure using hexane was established (Section 2.3) and the UV-Vis spectra of the extracts were recorded in the wavelength range of (200–400) nm. Figure 5 shows the UV-Vis spectra of the extracted core from O-MCs (Figure 5A) and S-MCs (Figure 5B). Absorption peaks centered at ~270 nm for the olive oil extracts and multiple peaks at 257, 267, and 278 nm for soybean oil extracts are found. In fact, it has been reported that the oxidation products of olive oil are found at 232 nm and 270 nm in cyclohexane solvent [53], while the oxidation products of soybean oil show the main absorption peaks at 232, 255, 265, and 270 nm, and two minor peaks in the regions of 290 and 310 nm [54]. These peaks, which are also observed in the hexane solutions of the respective oils (Figure S1A) permitted the construction of the calibration curves and the quantification of the entrapped oil, assuming that the oils do not undergo any further oxidation throughout all the processes applied (from the formation of microcapsules to the quantitative determination through UV-Vis). Thus, from the calibration curves at 270 nm and 268 nm, for olive oil (Figure S1B) and soybean oil (Figure S2B), respectively, the actual entrapment (P) of the two oils could be determined, and it is compared with the oil feed composition ($P_{Feed}$) in Table 2. As seen for all O-MCs and S-MCs studied, P was found to be very close to $P_{Feed}$, indicating that the oils were practically quantitatively encapsulated in the core of the microcapsules and, most importantly, successfully released through the extraction process.

#### 3.2.2. Castor Oil

In the case of castor oil-loaded MCs, our attempts to encapsulate high oil contents (50 and 60 wt.%) failed, since we observed macroscopic precipitation during polymerization. On the other hand, as previously reported, the preparation of microcapsules was successful for lower castor oil contents (20, 30 and 40 wt.%). However, we were not able to detect castor oil through UV-Vis spectrophotometry in the extracts of C-MCs(20), C-MCs(30), and C-MCs(40) (Figure S4), though we verified that castor oil dissolves in the organic solvent used for extraction, namely chloroform, and the calibration curve at 285 nm [64] was readily constructed (Figure S5).

**Figure 6:**
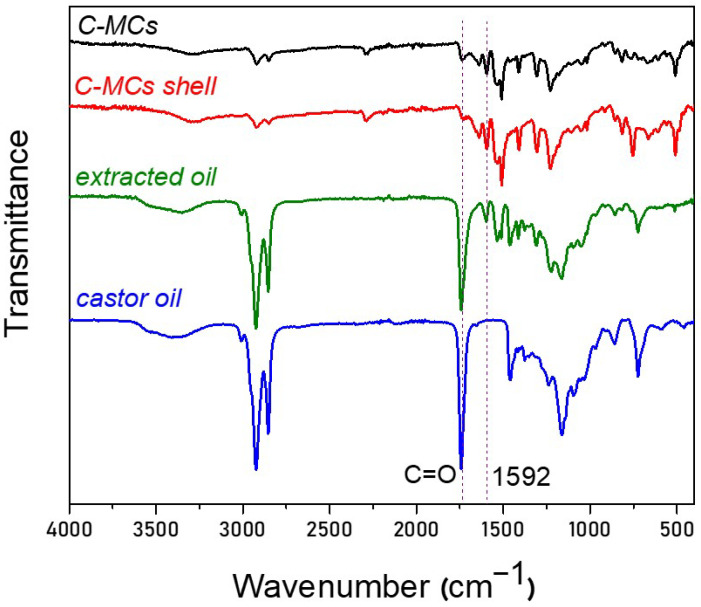
Spectra of C-MCs, precipitate (shell), supernatant (extracted core), and castor oil.

For further verification, the extracts after evaporation of the organic solvent were characterized through ATR-FTIR spectroscopy. As an example, Figure 6 shows the spectra of C-MCs(30), the precipitate (shell), and the respective dried extract. A weak peak at 2287 cm^−1^ of unreacted NCO groups is observed only in the spectrum of the shell, while the spectrum of the dried extract is very similar to that of the castor oil (see, for example, the characteristic peaks at 2700–2900 attributed to C-H groups, as well as the peak at 1737 cm^−1^, attributed to the carbonyl group of castor oil). Apparently, the quantity of castor oil, though present in the extract, is substantially low and it cannot be quantified through UV-Vis spectrophotometry.

**Figure 7:**
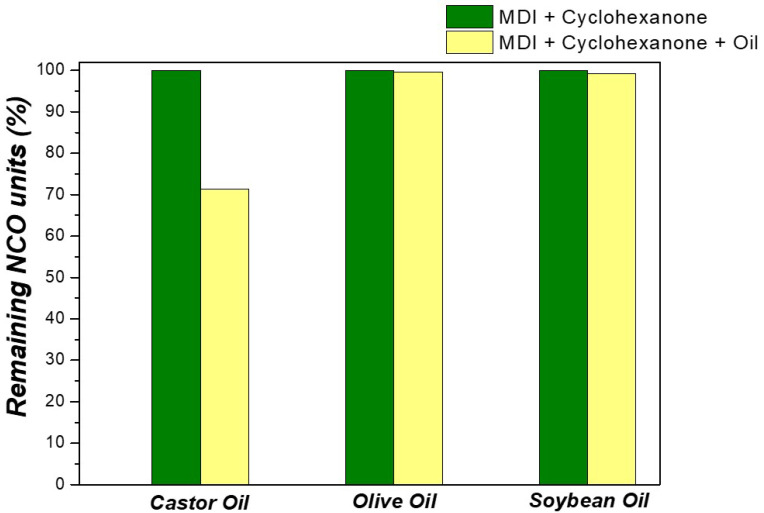
Remaining NCO units (%) in the solutions of (green bar) MDI in cyclohexanone (standard solution) and (yellow bar) MDI and each oil in cyclohexanone.

A reason for the aforementioned observation might be the possible reaction of the OH groups of the ricinoleic acid units contained in castor oil with the NCO groups of MDI [65,66]. To verify this possibility, we prepared mixtures that were identical to those used for the preparation of O-MCs(30), S-MCs(30), and C-MCs(30), and we titrated these mixtures according to the ASTM D2572 standard, after a time (1.5 h), similarly to that applied for the preparation of MCs. Figure 7 presents the content of isocyanate functional groups (NCO, %) remaining in the solution compared to the blank experiment (in the absence of any oil). For the blank experiment, as well as for the experiments in the presence of olive oil and soybean oil, the percentage of NCO is about 3.6%, which is in good agreement with the MDI feed composition. In contrast, in the case of castor oil, a ~30% decrease is observed, and the remaining NCO content is only ~2.6%. This decrease suggests a partial reaction of the OH groups of castor oil, with residual hydroxyl groups still being present in the system.

Since the hydroxyl content of castor oil (high content of ricinoleic acid) is significant compared to the other two oils (olive and soybean oils), the reactions—between hydroxyl groups (OH) and towards isocyanate groups (NCO)—consume a significant amount of castor oil during the microcapsule formation process, resulting in a decrease in the amount of free oil that can exert a protective effect. Due to this high chemical reactivity, a lower anti-corrosive performance of castor oil can be observed compared to other oils in the next study.

### 3.3. Self-Healing Properties and Anticorrosion Test

The potential self-healing property of water-based polyurethane coatings containing VO-MCs was explored. It was observed that microcapsules with low vegetable oil content are easily dispersed in water through gentle magnetic stirring; apparently, this is due to the presence of OH groups of the emulsifier in the shell of the microcapsules, providing high dispersibility in water. In contrast, the capsules with a high vegetable oil content were hardly dispersed in aqueous media. These can be dispersed, however, in organic solvents (such as ethanol, hexane, etc.). Since we avoided the use of organic solvents in our work, capsules with low vegetable oil content were used as additives for the study of the progress of the self-healing and anti-corrosion tests.

**Figure 8:**
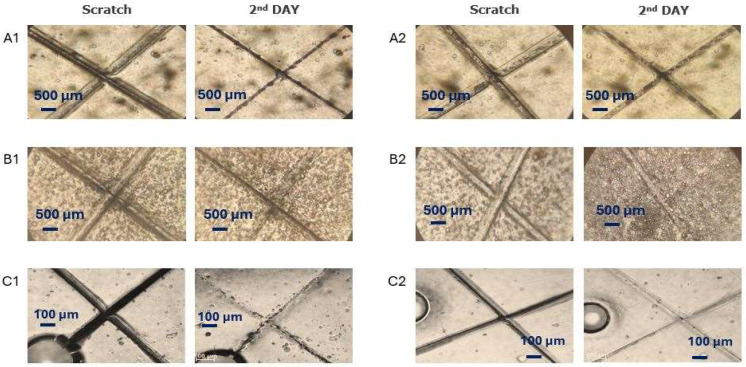
Progress of self-healing ability in films enriched with microcapsules loaded with the following: (**A1**,**A2**) olive oil, (**B1**,**B2**) soybean oil, and (**C1**,**C2**) castor oil, under conditions of partial humidity (**A1**,**B1**,**C1**) or controlled temperature of 60 °C (**A2**,**B2**,**C2**).

The aqueous microcapsules’ dispersion was added dropwise into polyurethane matrices to prepare self-healing coatings with VO-MCs as additives at the final concentration at 3.0 wt.%. The coatings were applied to Teflon. In fact, due to the low surface energy of Teflon [67], the ability of the aqueous mixture to spread on its surface is quite low, and the formed films (after water evaporation) were readily detached from the substrate. After scratching, the films were left under humid conditions or at 60 °C. The evolution of the scratches was followed by optical microscopy (Figure 8). It is evident that the most promising results are obtained after curing the VO-MCs films at 60 °C (Figure 8, right), as compared to the use of water as a trigger (Figure 8, left).

For the film of pure polyurethane (without microcapsules), the scratched surface can be efficiently healed under the conditions of partial humidity, as shown in Figure S6. This is quite reasonable, because the WPUs can intrinsically self-heal through a network of dynamic hydrogen bonds (H-bonds). In this case, the addition of a few drops of water is a key factor, because water acts as a trigger, softening the material and allowing for the polymer chains to move and reconnect [37,68,69,70]. For a clearer evaluation of these observations, the quantitative assessment of self-healing effectiveness was also performed, based on the decrease in the scratch width, as observed from the top. Indeed, our findings suggest that the healing effects observed under these conditions result mainly from the intrinsic dynamic behavior of the pure polyurethane matrix itself, while the released oil may be partially removed upon contact with the water. As shown in Figure S7, the decrease in the scratch width for the MCs-loaded films is similar to that found for the pure WPU film (~70%). On the other hand, when the pure WPU film is cured at 60 °C (well below the glass transition temperature of WPUs), the damaged surface is unable to practically intrinsically heal, and the decrease in the scratch width is marginal (~10%). In contrast, extrinsic self-healing is very pronounced for the VO-MCs-loaded films: the determined decrease in the scratch width is 74%, 97%, and 90% for O-MCs, S-MCs, and C-MCs, respectively. These data confirm that the impact of temperature on the self-healing mechanism is more pronounced, essentially enhancing the mobility and fluidity of vegetable oils and leading to more effective bridging and refilling of the damaged site.

For all kinds of MCs, after scratching, the VO-MCs within the film become ruptured, and the entrapped vegetable oil is directly released from the embedded microcapsules and reacts with oxygen to form a new film, healing the damaged area. The elevated temperature decreases the oil viscosity [71,72,73], enabling the penetration of the vegetable oils into the microscopic cracks or imperfections of the surface. Vegetable oil oxidation leads into the formation of oligopolymers of triacylglycerols, and the rate at which this happens under given conditions depends on the degree of unsaturation and the concentration of polyunsaturated fatty acids, as well as the presence of pro- or antioxidant compounds [74]. A temperature increase can also act like a trigger to accelerate oxidation; however, the temperature and time applied in this work during heating are milder than the ones required to induce the formation of oligopolymers [75,76]. It is expected that due to the low unsaturation level of the vegetable oils, the film formed is initially liquid and then gradually becomes more viscous and solid-like. The introduction of pro-oxidants in separate capsules could accelerate this transition.

**Figure 9:**
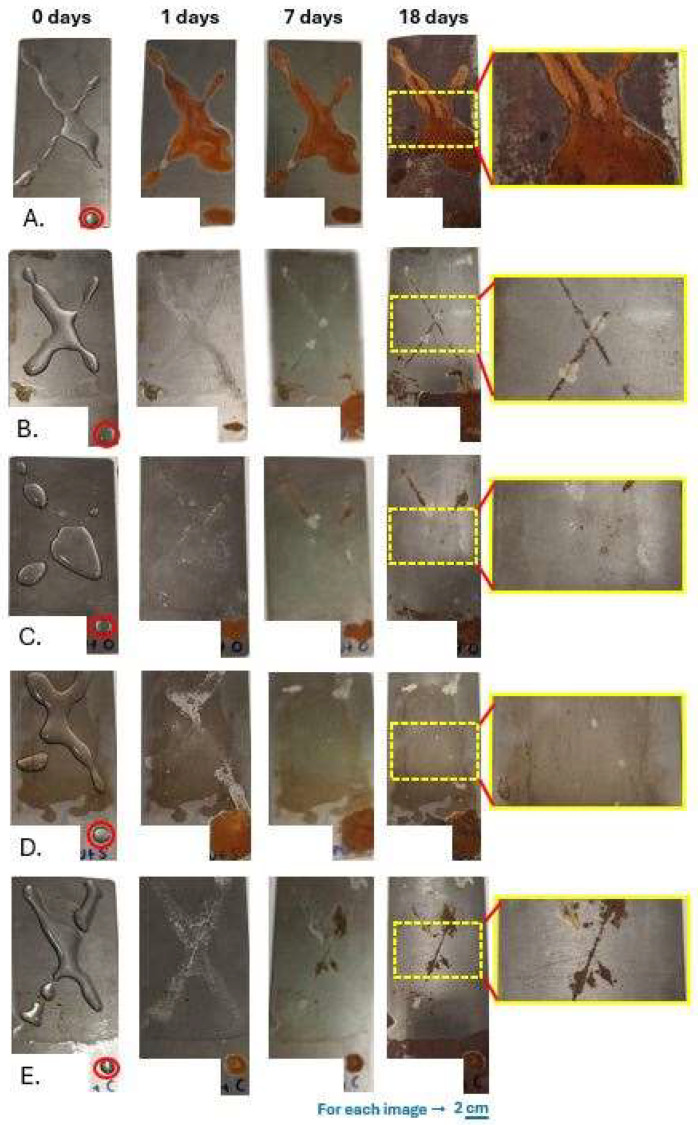
Optical images of corrosion protection of metallic plates, coated or not. Metal (**A**) without coating and with polyurethane coating: (**B**) neat, (**C**) loaded with O-MCs, (**D**) loaded with S-MCs, and (**E**) loaded with C-MCs. The 4th day is defined as the creation of the scratch. After remaining at 60 °C for 2 days, on the 7th day, the drops of neutral salt are added. Finally, the 8th day, the 14th day, and the 25th day correspond to the 1st day, 6th day, and 17th day, respectively, of metals, coated or not, under NSE.

The anticorrosion performance of the scratched coatings on a metal substrate was studied by a visual observation. As illustrated in Figure 9, the neat metal exhibits poor resistance to corrosion at the first exposure to neutral salt. Corrosion is clearly observable already on the first day, after applying the drop of the salt solution. After 18 days, rust is observed on the entire metal surface, not just in the area of the scratch, due to the absence of a coating material. The pure polyurethane coating exhibits a medium resistance to corrosion. On the seventh day, scratches from the rust that have penetrated the gap created between the two sides of the coating material begin to be visible. On the 18th day, the presence of rust is evident at the points of the scratches, forming the letter X.

For the self-healing coating, loaded with VO-MCs, corrosion is not as severe as the neat polyurethane coating. The self-healing coatings enriched with O-MCs show no signs of rusting on the first day. After seven days, it is evident that some sections (upper metal area) in the overall scratch have been affected by the addition of the salt, while other sections (lower metal area) seem to have healed and therefore do not allow rust to form. This is confirmed, as the metal has not corroded further on the 18th day. The self-healing coatings loaded with S-MCs showed no visible rust effects up to the 18th day. In fact, the scratches appear to be completely healed, and therefore, the S-MCs offer particularly good anticorrosion properties for the metal. In contrast, the coatings loaded with C-MCs show rust-spots from the first day, while corrosion continues to expand over the days.

In summary, these results are comparable and essentially parallel to the self-healing progress in a closed chamber with a controlled temperature (60 °C). The incorporation of S-MCs significantly enhances the self-healing capability of the polyurethane film, while O-MCs contribute only partially to this improvement. On the contrary, the use of C-MCs while showing self-healing to a large extent in the film, shows poor corrosion resistance from the first day of the stay in the NSE in the anti-corrosion test. This is likely due to the low content of double bonds in this particular oil, but also to the intense reactivity of OH-groups to react with the NCO-groups of the organic phase, leaving a small percentage to act as a self-healing agent. To sum up, the self-healing coating exhibits good long-term anti-corrosion properties, mainly using S-MCs.

## 4. Conclusions

This study presents an approach for the preparation of self-healing polyurethane coatings activated by thermal stimuli, through the encapsulation of vegetable oils in polyurea microcapsules. Encapsulation of vegetable oils was successfully achieved through emulsion polymerization, with the type and content of the oil being key factors for the performance, characteristics, and failure of the final system. All types of microcapsules prepared were dispersed in an aqueous polyurethane matrix, ensuring their distribution in the final material. The core idea is that encapsulation leads to the immediate release of the healing agent into the damaged area, while ensuring self-healing of the surface and the ability to protect metal surfaces from corrosion. The key factor concerns the process of releasing vegetable oils within the WPU. The microcapsules are designed to crack under mechanical pressure, allowing for the oil to be released directly into the damaged area. Temperature significantly affects the viscosity of the oil. The oil can become more fluid when temperature increases locally. Then, the oil can fill the scratch and polymerize or oxidize, healing the scratch. Regarding the healing mechanism, a marked correlation was observed, depending on the type and content of the oil, as well as with the environmental conditions that allow for the microcapsule to be activated and the oil to act optimally. In the present study, we focused on the visual/macroscopic assessment of self-healing and the anti-corrosion performance of our materials. For a complete evaluation of these properties, mechanical and electrochemical impedance spectroscopy studies of the most promising materials is considered to be essential, as a further step.

In the present study, thermal activation at 60 °C was selected to demonstrate the proof-of-concept. While a lower temperature can also be studied, the present conditions are representative of several environments with increased operating temperatures (industrial tubing, external metal substrates in summer, thermal rise due to friction/sunlight, etc.).

## Figures and Tables

**Table 1:** Vegetable oil encapsulation attempts into polyurea MCs at various wt.% of oil in the organic phase, $P_{Feed}$ (Equation (1)). The symbol (✓) denotes the preparation of a stable emulsion throughout the reaction (3 h), while (✕) denotes its precipitation.

Vegetable Oil	$\mathrm{wt}.\%\;\mathrm{Oil}\;\mathrm{in}\;\mathrm{Organic}\;\mathrm{Phase},\;P_{Feed}$					
	20	30	40	50	60	70
Olive oil				✓	✓	✓
Soybean oil			✓	✓	✓	
Castor oil	✓	✓	✓	✕	✕	

**Table 2:** Comparison of the actual (P) and feed ($P_{Feed}$) composition in olive oil and soybean oil, encapsulated in the core of the respective microcapsules.

	A	B	C	D	E	F
Code	O-MCs(50)	O-MCs(60)	O-MCs(70)	S-MCs(40)	S-MCs(50)	S-MCs(60)
$P_{Feed}$	50%	60%	70%	40%	50%	60%
P	44%	60%	66%	38%	51%	58%

## Data Availability

The original contributions presented in this study are included in the article/Supplementary Material. Further inquiries can be directed to the corresponding author.

## Supplementary Materials

The following supporting information can be downloaded at: https://www.mdpi.com/article/10.3390/polym17233184/s1. Figures S1, S2 and S5: (A) UV-Vis spectra of standard solutions and (B) calibration curve of olive and soybean oil in hexane and castor oil in chloroform, respectively; Figure S3: ATR-FTIR spectra of (a) O-MCs(50), O-MCs(60), and O-MCs(70), (b) C-MCs(20), C-MCs(30), and C-MCs(40) and (c) S-MCs(40), S-MCs(50), and S-MCs(60); Figure S4: UV-Vis spectra of extracted core for C-MCs; Figure S6: Progress of self-healing ability in pure polyurethane film under conditions of partial humidity (A) or controlled temperature, 60 °C (B). Figure S7: Decrease of scratch width (%) on films: pure WPU and WPU loaded with O-MCs, S-MCs and C-MCs which was triggered under conditions of partial humidity or controlled temperature, 60 °C.
