# Supplementary material for: Vegetable-Oil-Loaded Microcapsules for Self-Healing Polyurethane Coatings
Source: Polymers (Basel). 2025 Nov 29;17(23):3184. doi: 10.3390/polym17233184 (PMC12693790; doi:10.3390/polym17233184)
Supplement: Supplementary file 1 [file polymers-17-03184-s001.zip › polymers-4001912-supplementary.pdf]

Vegetable-Oil-Loaded Microcapsules for Self-Healing Polyurethane Coatings

Efterpi Avdeliodi [1], Sofia Derizioti [1], Ioanna Papadopoulou [1], Aikaterini Arvaniti [1], Kalliopi Krassa [2], Eleni P. Kalogianni [3], Joannis K. Kallitsis [1] and Georgios Bokias [1,*]

[1] Department of Chemistry, University of Patras, 26504 Patras, Greece; chem3577@ac.upatras.gr (E.A.); up1066326@ac.upatras.gr (S.D.); up1073739@ac.upatras.gr (I.P.); up1087065@ac.upatras.gr (A.A.); kallitsi@upatras.gr (J.K.K.)
[2] Megara Resins Fanis S.A., 38th KM New National Rd. Athens-Corinth, 19100 Megara Attikis, Greece; p.krassa@megararesins.com
[3] Department of Food Science and Technology, International Hellenic University, 57400 Thessaloniki, Greece; elekalo@ihu.gr
* Correspondence: bokias@upatras.gr; Tel.: +30-2610-997102

Supplementary Information

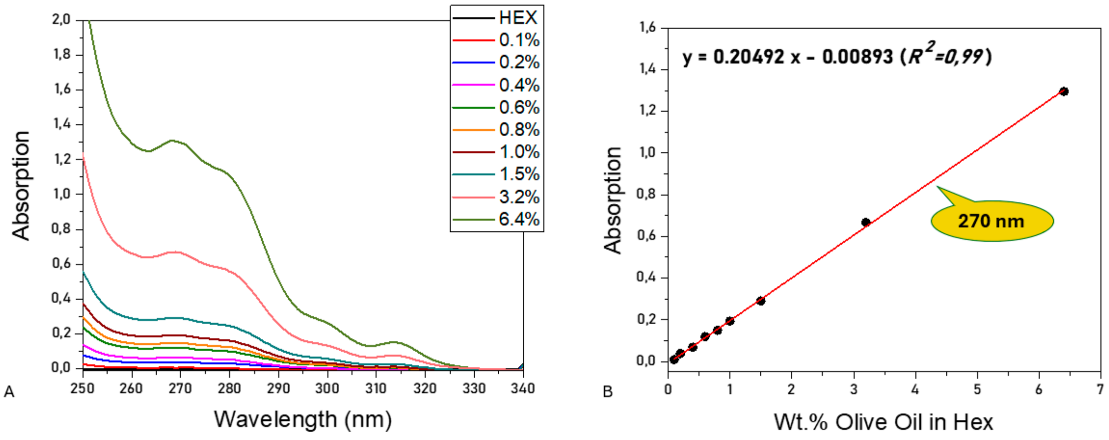

Figure S1. (A) UV-Vis spectra of standard solutions of olive oil in hexane. (B) Calibration curve of olive oil in hexane.

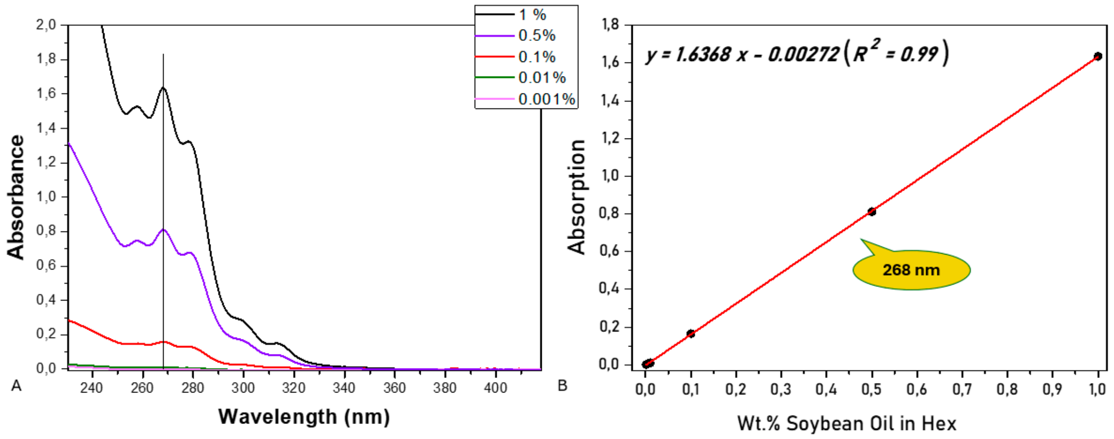

Figure S2. (A) UV-Vis spectra of standard solutions of soybean oil in hexane. (B) Calibration curve of soybean oil in hexane.

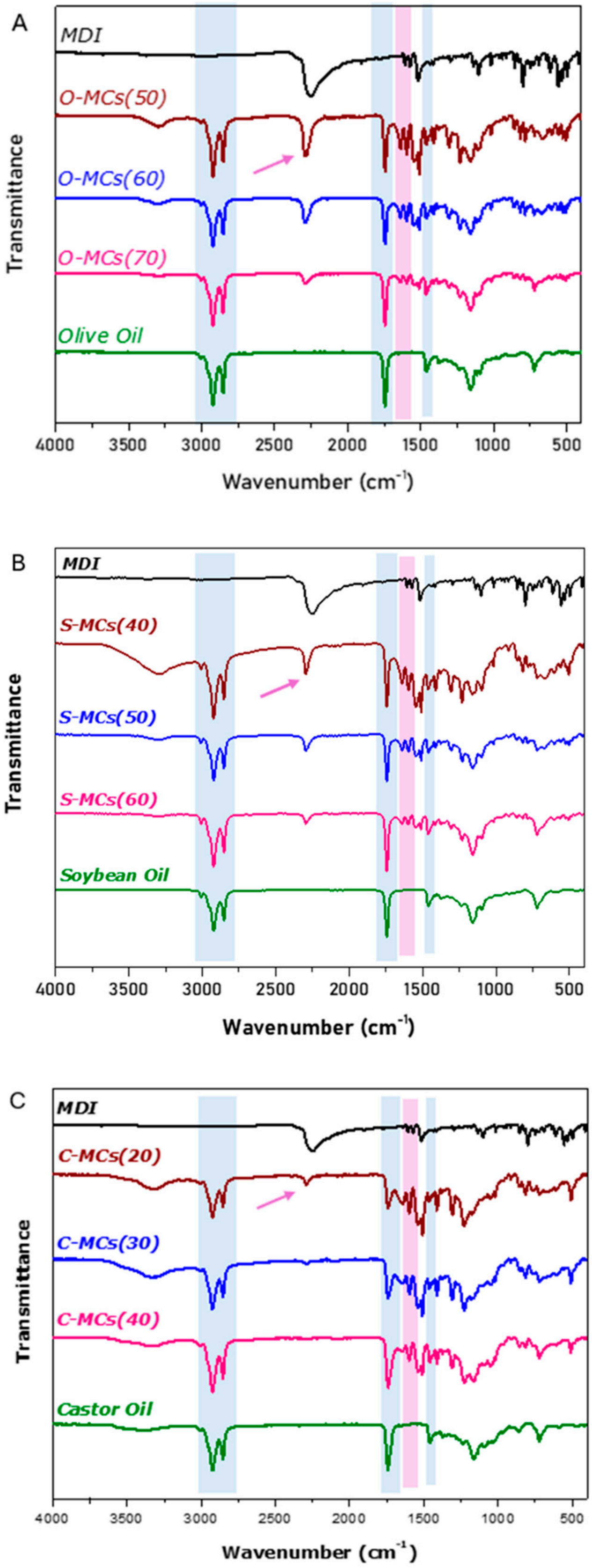

Figure S3. ATR-FTIR spectra of (a) O-MCs(50), O-MCs(60) and O-MCs(70), (b) S-MCs(40), S-MCs(50) and S-MCs(60) and (c) C-MCs(20), C-MCs(30) and C-MCs(40).

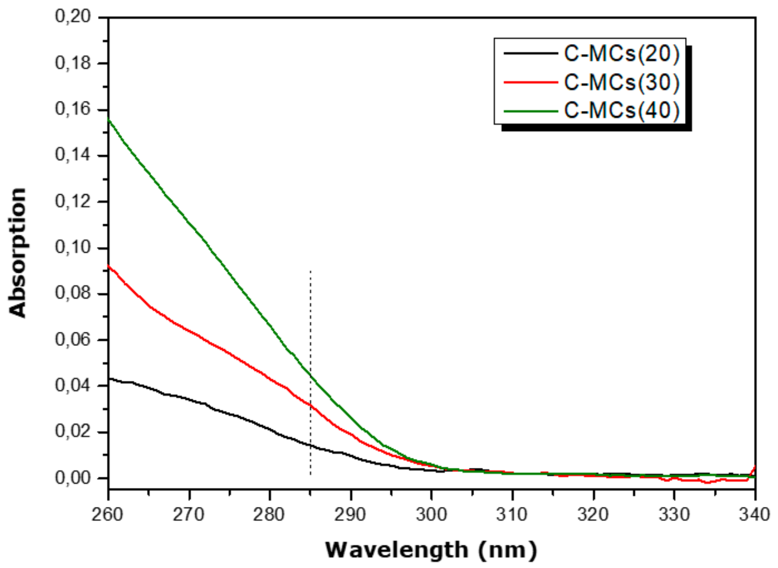

Figure S4. UV-Vis spectra of extracted core for C-MCs.

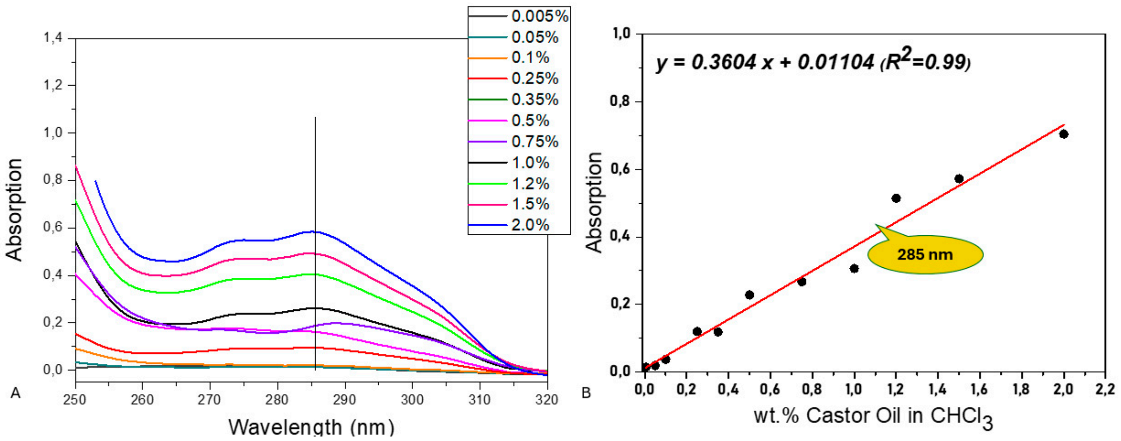

Figure S5. (A) UV-Vis spectra of standard solutions of castor oil in chloroform. (B) Calibration curve of castor oil in chloroform.

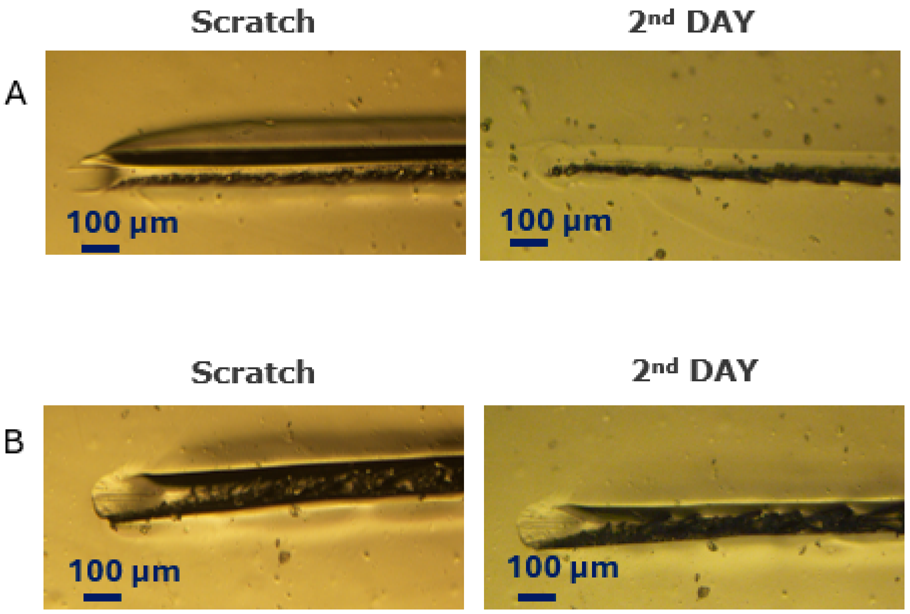

Figure S6. Progress of self-healing ability in pure polyurethane film under conditions of partial humidity (A) or controlled temperature, 60 ℃ (B).

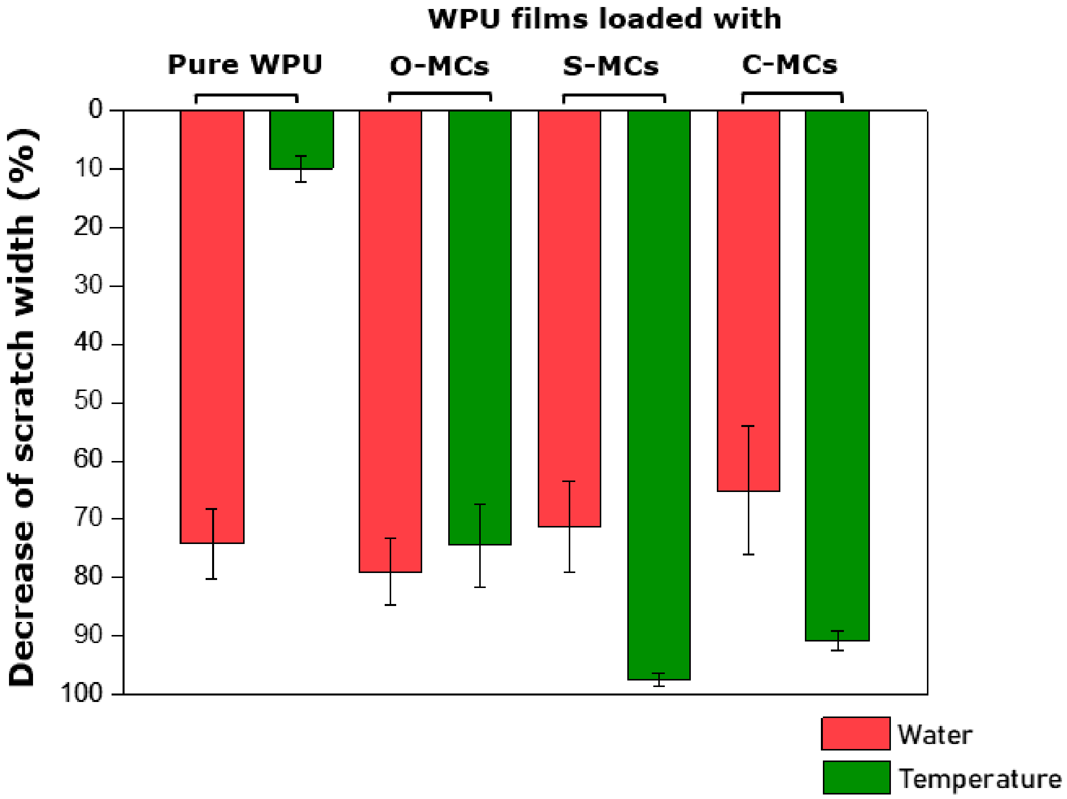

Figure S7. Decrease of scratch width (%) on films: pure WPU and WPU loaded with O-MCs, S-MCs and C-MCs. Self-healing phenomenon was triggered film under conditions of partial humidity or controlled temperature, 60 ℃.
